# Slide tracheoplasty versus tracheal resection anastomosis for acquired laryngotracheal stenosis: a prospective randomized controlled trial

**DOI:** 10.1007/s00405-026-10296-8

**Published:** 2026-05-21

**Authors:** Eslam Hamed Abdou, Mohamed Elkahwagi, Ahmed Abdelfattah Elsobki, Mohamed Elshaer

**Affiliations:** Department of Otolaryngology–Head and Neck Surgery, Faculty of Medicine, Mansoura University, Mansoura, Egypt

**Keywords:** Laryngotracheal stenosis, Slide tracheoplasty, Tracheal resection, Airway reconstruction, Randomized controlled trial, Functional outcomes

## Abstract

**Background:**

Tracheal resection with end-to-end anastomosis remains the standard surgical treatment for acquired high-grade laryngotracheal stenosis. Slide tracheoplasty has emerged as an alternative reconstructive technique that enlarges airway caliber while preserving tracheal length and vascularity, yet direct comparative evidence in acquired stenosis is limited.

**Objective:**

To compare the clinical, surgical, and functional outcomes of slide tracheoplasty versus tracheal resection with end-to-end anastomosis in patients with acquired laryngotracheal stenosis.

**Methods:**

In this prospective pilot randomized controlled trial, 30 patients with grade II–IV acquired tracheal stenosis were allocated 1:1 to slide tracheoplasty (n = 15) or tracheal resection with end-to-end anastomosis (n = 15). Randomization was performed using a computer-generated sequence with variable block sizes, and baseline characteristics were assessed for group comparability. The primary outcome was surgery-specific success, defined as successful decannulation without need for revision open surgery within 6 months. Secondary outcomes included operative time, decannulation time, complications, restenosis, postoperative interventions, hospital stay, dyspnea (MRC scale), voice quality (VHI-10), and swallowing function (GUSS, PAS). Analyses followed the intention-to-treat principle.

**Results:**

A total of 30 patients were randomized and completed follow-up. Baseline characteristics were comparable between groups. Surgery-specific success was high in both arms (slide 93.3% vs resection 86.7%; RR 1.08, 95% CI 0.85–1.37; p > 0.99). Slide tracheoplasty required longer operative time (152.3 ± 15.7 vs 134.0 ± 17.5 min; p = 0.005) but resulted in earlier decannulation (12.3 ± 3.2 vs 16.3 ± 5.0 days; p = 0.013) and fewer postoperative balloon dilatations (13.3% vs 40%). Hospital stay and overall complication rates were similar. Both procedures significantly improved dyspnea and voice outcomes; however, postoperative MRC and VHI-10 scores favored slide tracheoplasty (p < 0.05). Early postoperative swallowing impairment occurred more frequently after resection, with full recovery in both groups by one month. No mortality occurred.

**Conclusions:**

In this pilot randomized controlled trial, both slide tracheoplasty and tracheal resection with end-to-end anastomosis were associated with high success rates and acceptable safety profiles in acquired laryngotracheal stenosis. Slide tracheoplasty demonstrated comparable success and was associated with earlier decannulation and a trend toward improved short-term functional recovery; however, findings should be interpreted cautiously given the exploratory nature of the study. Larger multicenter trials are required to confirm these preliminary observations.

**Trial registration:**

Clinical Trials.gov (NCT06917222).

## Introduction

Acquired laryngotracheal stenosis remains a challenging clinical entity associated with substantial respiratory and functional morbidity, including dyspnea, impaired phonation, dysphagia, and prolonged tracheostomy dependence. Post-intubation and post-tracheostomy injuries are the most frequent causes, typically resulting in circumferential fibrosis and fixed airway narrowing that often require definitive surgical reconstruction rather than repeated endoscopic interventions [1, 2].

Tracheal resection with end-to-end anastomosis has historically been regarded as the standard operative treatment for short-segment high-grade stenosis, with reported success rates exceeding 85–95% in experienced centers [3, 4]. Nevertheless, circumferential resection may introduce anastomotic tension, reduce tracheal length, and alter laryngotracheal mechanics, potentially influencing postoperative recovery and functional outcomes such as swallowing and voice quality [3, 5].

Slide tracheoplasty was originally developed for long-segment and congenital tracheal stenosis and achieves airway enlargement by longitudinal division and sliding reconstruction of the native trachea, thereby preserving vascular supply, maintaining structural integrity, and reducing anastomotic tension [5]. Contemporary series have demonstrated excellent safety and durable patency using this technique, and its application has expanded beyond congenital disease to selected acquired lesions [6, 7]. Despite these promising results, direct comparative evidence between slide tracheoplasty and conventional tracheal resection with end-to-end anastomosis for acquired stenosis remains limited.

Importantly, most published studies consist of retrospective case series without control groups, restricting meaningful evaluation of comparative effectiveness and patient-centered outcomes. Functional measures including time to decannulation, dyspnea severity, voice quality, and swallowing safety are increasingly recognized as critical determinants of postoperative recovery and quality of life, yet these parameters are rarely assessed systematically in airway reconstruction studies [2].

Accordingly, we conducted a prospective randomized controlled trial to compare slide tracheoplasty and tracheal resection with end-to-end anastomosis in patients with acquired laryngotracheal stenosis. We hypothesized that slide tracheoplasty would provide comparable surgical success while facilitating earlier decannulation and early functional outcomes without increasing perioperative morbidity.

## Materials and methods

### Study design

A prospective, single-blinded, randomized controlled trial was conducted at Mansoura University Hospital, a tertiary referral center. The study aimed to compare the clinical, functional, and surgical outcomes of slide tracheoplasty versus tracheal resection with end-to-end anastomosis in patients with acquired laryngotracheal stenosis. The trial was conducted in accordance with the CONSORT 2010 guidelines and the Declaration of Helsinki. The study was approved by the Mansoura Faculty of Medicine Institutional Research Board (MFM-IRB) (MD.25.02.956) and was prospectively registered at ClinicalTrials.gov (NCT06917222) prior to patient enrollment. Written informed consent was obtained from all participants or their legal guardians before enrollment in the study. Patient confidentiality and data privacy were strictly maintained throughout the study.

### Study population

Patients of all ages with symptomatic high-grade II (after failed endoscopic interventions) and grade III/IV acquired tracheal stenosis suitable for either slide tracheoplasty or tracheal resection with end-to-end anastomosis were eligible. Exclusion criteria included congenital tracheal stenosis, glottic involvement, malignant airway lesions, stenosis deemed surgically unreachable due to excessive length or severe anatomical distortion, revision tracheal surgery, refusal of consent, and inability to complete the six-month follow-up period.

### Sample size calculation

Due to absence of prior RCTs comparing these interventions in acquired stenosis, effect-size estimates were unavailable. As a pilot trial, a pragmatic sample of 15 patients per group was selected to assess feasibility, safety, and generate preliminary effect-size estimates for future trials. This sample allows estimation of standard deviations, recruitment rates, and variability in primary and secondary outcomes.

### Randomization

Thirty patients were randomly assigned in a 1:1 ratio to one of two treatment arms: Slide Tracheoplasty Group (n = 15) and Tracheal Resection and End-to-End Anastomosis Group (n = 15) using a computer-generated random sequence. Given the pilot nature and limited sample size, formal stratified randomization was not performed. Instead, block randomization with variable block sizes was used to ensure balanced allocation. Baseline comparability between groups was assessed across key clinical variables to confirm absence of clinically meaningful imbalance.

### Blinding

The study followed a single-blinded design in which postoperative outcome assessors and speech-language therapists assessing swallowing and voice outcomes, were blinded to treatment allocation. Patient-reported outcomes were collected using validated instruments (MRC, VHI-10). Patients and operating surgeons were not blinded due to the nature of the intervention. Measures were taken to minimize detection bias through standardized assessment protocols and predefined outcome measures.

### Preoperative evaluation

Preoperative variables included age, sex, body mass index (BMI), comorbidities, history of prior balloon dilatation or airway surgery, etiology of tracheal stenosis, and the length and the grade of the stenotic segment.

### Outcome measures

All primary and secondary outcomes were predefined before patient enrollment and were not modified during the course of the study.

The primary outcome was surgery-specific success, defined as decannulation with a symptom-free airway and no requirement for revision open surgery within 6 months. Up to two postoperative endoscopic dilatations were permitted and not considered treatment failure, provided successful decannulation and symptom resolution were achieved. We defined failure as persistent symptoms or obstructive airway lesions despite surgery, the requirement for more than two endoscopic interventions, and/or failure of decannulation.

Secondary endpoints included symptom improvement that assessed using the Medical Research Council (MRC) Breathlessness Scale, with improvement defined as a reduction in postoperative MRC grade compared to baseline; mortality; immediate or delayed postoperative complications; duration of the surgery; need for tracheostomy, time to decannulation; requirement for open or endoscopic airway revision surgery; and total postoperative hospital length of stay.

### Functional outcome assessment

Swallow assessments were carried out by a specialist Speech and Language Therapist and involved the following: Gugging Swallowing Screen (GUSS) was conducted according to Bengisu et al., [8]. Scores ranged from 0 to 20 and were categorized into four severity levels from severe dysphagia to normal swallowing function. Fiberoptic Endoscopic Evaluation of Swallowing (FEES) with Penetration–Aspiration Scale (PAS): Aspiration severity was graded using the 8-point PAS described by Rosenbek et al., [9].Voice quality was assessed using the Voice Handicap Index-10 (VHI-10) questionnaire. Scores > 11 were considered indicative of clinically significant voice impairment.

### Follow-Up

All patients were followed for a minimum of 6 months postoperatively, with scheduled clinical, endoscopic, and functional evaluations. All outcomes were assessed at predefined times points, including early postoperative assessment, one-month follow-up and six-month follow-up.

### Surgical techniques

**Fig. 1:**
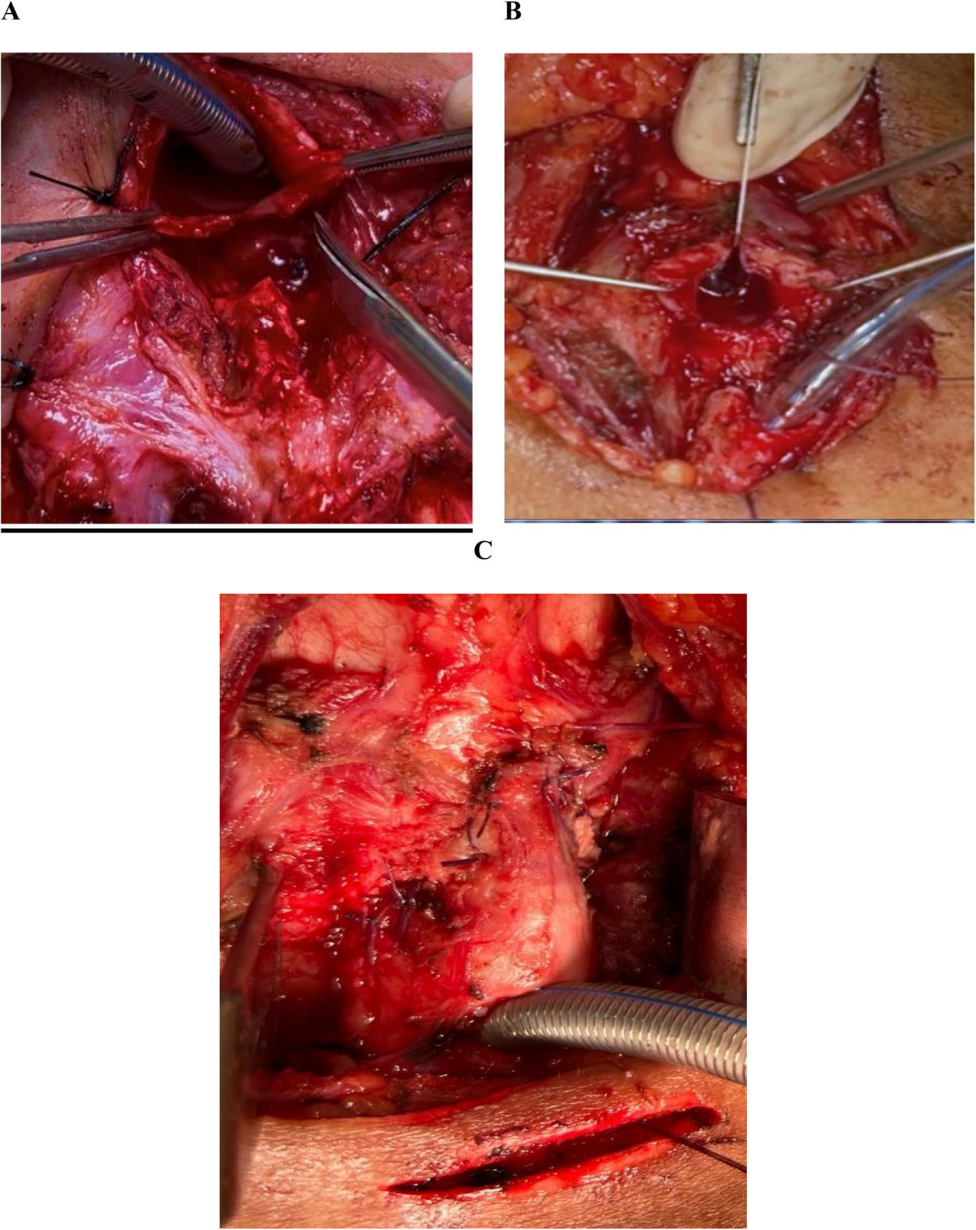
Slide tracheoplasty technique**.** (**A**) Cutting the posterior wall of the distal tracheal segment. (green arrow),(**B**) Cutting the anterior wall of the proximal tracheal segment. (black arrow),(**C**) Anastomosed using an interrupted suture. All panels are presented in the same orientation

**Fig. 2:**
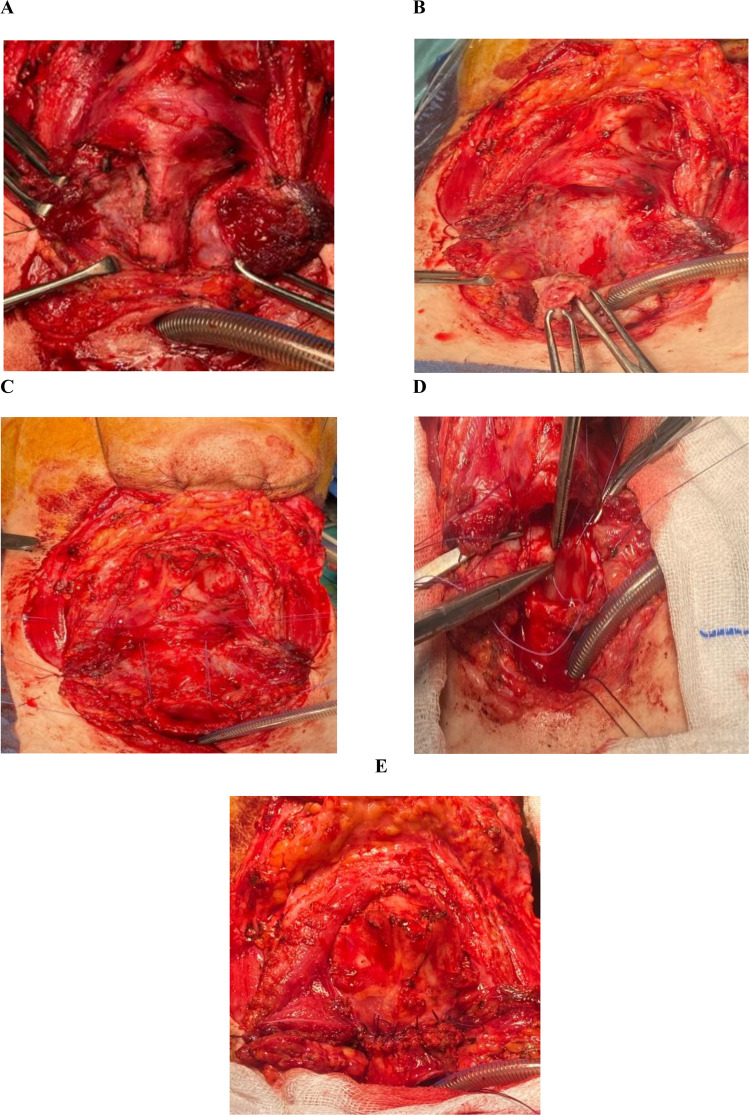
Operative Technique Illustration of tracheal resection with end to end anastomosis. (A) Dissection of the stenotic part of the trachea without identifying the RNLs. (black arrow demonstrates the stenotic segment), (yellow arrow demonstrates the divided thyroid gland) (**B**) Resection of the stenotic segment.(black star). (**C**) Two posterolateral sutures used to help reduce tension (**D**) The posterior membranous wall was addressed first (blue star). (**E**) Cartilaginous wall approximation completing the reconstruction.

All procedures were performed by experienced airway surgeons using standardized operative protocols. Slide Tracheoplasty: Performed by transecting the stenotic segment transversely at its midpoint, followed by longitudinal incisions on the anterior wall of the proximal segment and the posterior wall of the distal segment, allowing sliding and enlargement of the airway lumen before reconstruction [6]. Tracheal Resection with End-to-End Anastomosis: Involved complete excision of the stenotic tracheal segment followed by primary end-to-end anastomosis using tension-free suturing techniques [10]. A double-stage procedure was defined as postoperative maintenance of a tracheostomy with planned decannulation at a later stage; a single-stage procedure omitted postoperative tracheostomy. Figures. 1 and 2.

### Statistical analysis

Statistical analysis was performed using the Statistical Package of Social Science (SPSS) program for Windows, version 26. Continuous variables were presented as mean ± standard deviation or median (interquartile range), while categorical variables were presented as frequencies and percentages. Between-group comparisons were performed using appropriate parametric or non-parametric tests. Normality was assessed using Shapiro–Wilk testing. Continuous variables were compared using independent t-test or Mann–Whitney U as appropriate. Categorical variables were analyzed using Fisher’s exact or χ^2^ test. A p-value < 0.05 was considered statistically significant. Given the small sample size and use of exact tests, some p-values approached unity and are reported as p > 0.99. A post hoc sensitivity interpretation using a stricter definition of surgical success (excluding any postoperative dilatation) was considered descriptively.

## Results

A total of 30 patients with acquired laryngotracheal stenosis were included in the final analysis. Patients were randomly assigned to either the slide tracheoplasty group (n = 15) or the tracheal resection with end-to-end anastomosis group (n = 15). All patients completed the 6-month follow-up and were included in the intention-to-treat analysis. No losses to follow-up or protocol deviations occurred.

### Baseline characteristics

The slide tracheoplasty and tracheal resection with end-to-end anastomosis groups were well matched at baseline demographic and clinical characteristics (Table 1). There were no statistically significant differences between groups regarding age, sex distribution, body mass index, comorbidity profile, stenosis length, or etiology of laryngotracheal stenosis (all p > 0.05). Effect-size estimates indicated small to negligible differences (Cohen’s d < 0.2 for continuous variables; RR ~ 1 for categorical). Similarly, no significant differences were observed in stenosis grade distribution or the number of preoperative balloon dilatations (p = 0.45 and p = 0.52, respectively).

**Table 1:** Baseline demographic and clinical characteristics of the study groups

Characteristic	Resection (n = 15)	Slide (n = 15)	p-value	Effect size (95% CI)
Age, years (mean ± SD)	16.7 ± 5.4	17.7 ± 8.2	0.72†	d = 0.14 (−0.63, 0.91)
Median (IQR)	17 (14–20)	17 (15–23)	–	
Age category				
– Pediatric (< 18) n (%)	9 (60%)	9 (60%)	> 0.99‡	RR = 1.00 (0.54–1.85)
– Adult (≥ 18) n (%)	6 (40%)	6 (40%)	> 0.99‡	
Sex				
– Male n (%)	12 (80%)	12 (80%)	> 0.99‡	RR = 1.00 (0.65–1.55)
– Female n (%)	3 (20%)	3 (20%)	> 0.99‡	
BMI (mean ± SD)	25.3 ± 2.8	24.7 ± 3.2	0.76†	d = 0.20 (−0.57, 0.97)
Any comorbidity n (%)	7 (46.7%)	5 (33.3%)	0.71‡	RR = 0.71 (0.25–2.01)
Stenosis length, cm				
Mean ± SD	6.28 ± 0.90	6.03 ± 0.78	0.4†	d = 0.29 (−0.48, 1.06)
Median (IQR)	6.6 (5.4–7.1)	5.6 (5.2–6.5)	–	
Etiology				
– Post-intubation	12 (80.0%)	12 (80.0%)	–	
– Post-tracheostomy	2 (13.3%)	2 (13.3%)	–	
– Cervical trauma	1 (6.7%)	1 (6.7%)		
Post-intubation/tracheostomy/Trauma	12/2/1	12/2/1	> 0.99‡	
Stenosis grade (II/III/IV)	5/6/4	2/9/4	0.45‡	RR = 1.10 (0.68–1.78)
– Grade II	5 (33.3%)	2 (13.3%)		
– Grade III	6 (40.0%)	9 (60.0%)		
– Grade IV	4 (26.7%)	4 (26.7%)		
Preoperative balloon dilatations				
Mean ± SD	0.87 ± 0.83	1.07 ± 0.88	0.52‡	
Median (IQR)	1 (0–2)	1 (0–2)		
≥ 1 dilation	9 (60.0%)	10 (66.7%)	0.71†	RR = 1.11 (0.71–1.73)

† Independent-samples t-test.

‡ Fisher’s exact test.

### Operative details and surgical outcomes

Slide tracheoplasty was associated with a significantly longer operative time compared with tracheal resection with end-to-end anastomosis (152.3 ± 15.7 vs 134.0 ± 17.5 min; d = 1.10, p = 0.005, 95% CI for mean difference 7.2–33.4 min). However, patients undergoing slide tracheoplasty achieved significantly earlier decannulation (12.3 ± 3.2 vs 16.3 ± 5.0 days; d = 0.97, p = 0.013, 95% CI 1.1–7.5 days). Hospital stay was shorter in the slide group, although the difference did not reach statistical significance. (16.7 ± 3.7 vs 20.4 ± 6.5 days; d = 0.69, p = 0.068). Single-stage procedures were more frequently achieved in the slide tracheoplasty group, although this did not reach statistical significance (86.7% vs 73.3%; RR = 1.18, 95% CI 0.85–1.63, p = 0.65).

**Fig. 3:**
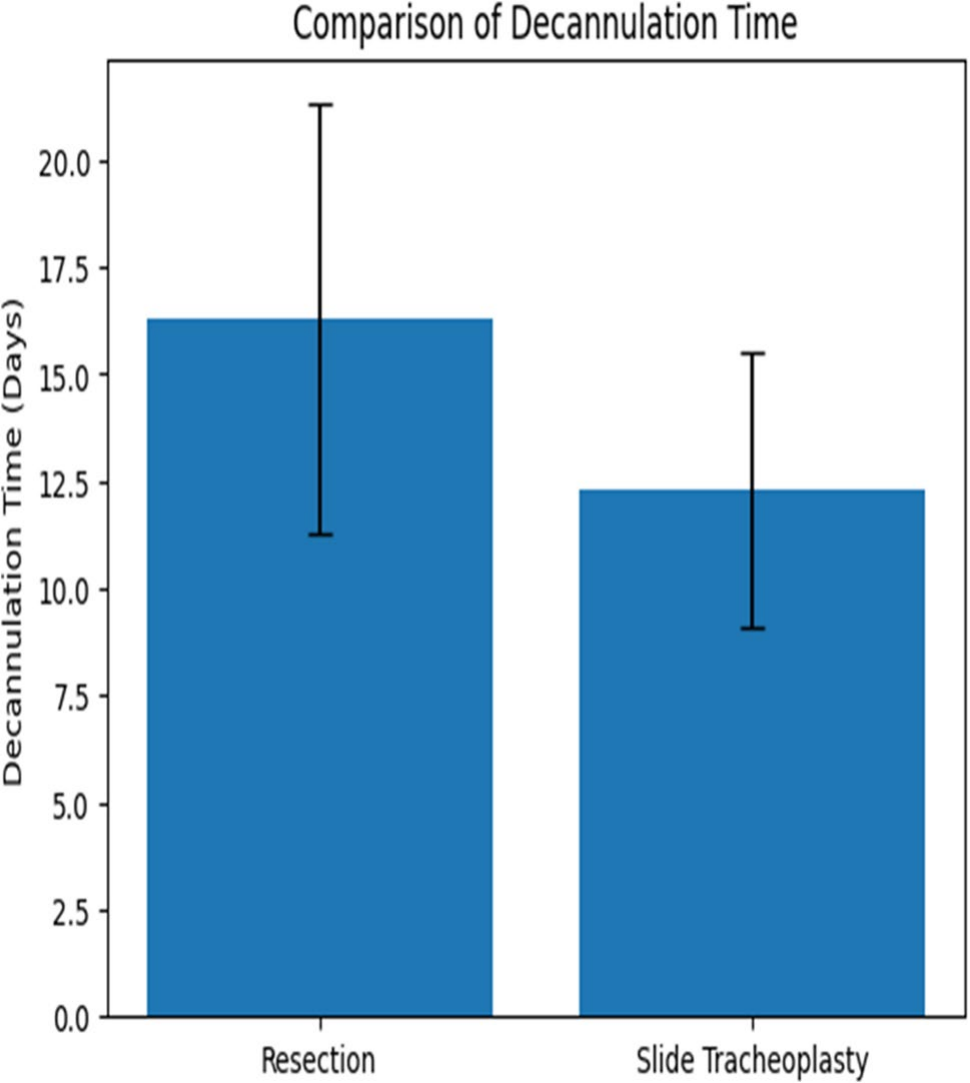
Comparison of Decannulation Time Between Surgical Techniques**.** Mean time to decannulation (± SD) in both groups. Slide tracheoplasty demonstrated shorter decannulation time (p = 0.013)

Overall complication rates were comparable between groups, with no statistically significant difference in restenosis or overall adverse events (2 in slide tracheoplasty group (13.3%) and 3 in resection with end-to-end anastomosis group (20%), RR = 0.67, 95% CI 0.13–3.44, p = 0.63). Restenosis was observed in 1 patient per group (6.7%; RR = 1.00, 95% CI 0.07–14.5, p > 0.99). Effect size estimates suggested a clinically meaningful reduction in the requirement for postoperative balloon dilatation in the slide tracheoplasty group (13.3% vs 40%; RR = 0.33, 95% CI 0.08–1.39, p = 0.12), despite not reaching statistical significance. This represents a moderate effect size suggesting a clinically meaningful reduction despite lack of statistical significance. Risk ratio analysis was associated with comparable surgery-specific success between techniques (RR 1.08, 95% CI 0.85–1.37). No mortality was recorded in either group (Table 2), (Fig. 3).

**Table 2:** Primary and secondary outcomes according to surgical technique

Outcome	Resection (n = 15)	Slide (n = 15)	p-value	Effect size (95% CI)
Surgery duration, mean ± SD (min)	134.0 ± 17.5	152.3 ± 15.7	0.005	*d* = 1.10 (7.2–33.4)
Decannulation time, mean ± SD (days)	16.3 ± 5.0	12.3 ± 3.2	0.013	*d* = 0.97 (1.1–7.5)
Hospital stay, mean ± SD (days)	20.4 ± 6.5	16.7 ± 3.7	0.068	*d* = 0.69 (−0.5–8.2)
Single stage surgery, n (%)	11 (73.3%)	13 (86.7%)	0.65‡	RR = 1.18 (0.85–1.63)
Any complication, n (%)	3 (20%)	2 (13.3%)	0.63	RR 0.67 (0.13–3.44)
Restenosis, n (%)	1 (6.7%)	1 (6.7%)	> 0.99‡	RR 1.00 (0.07–14.5)
Balloon dilations, median (range)	0 (0–4)	0 (0–4)	0.070	
≥ 1 postoperative balloon dilation, n (%)	6 (40%)	2 (13.3%)	0.12‡	RR 0.33 (0.08–1.39)
Surgery-specific success, n (%)	13 (86.7%)	14 (93.3%)	> 0.99‡	RR 1.08 (0.85–1.37)
Mortality, n (%)	0 (0%)	0 (0%)	—	—

‡ Fisher’s exact test.

Continuous variables are reported with Cohen’s d; categorical variables with risk ratios (RR) and 95% confidence intervals.

### Functional outcomes

Both groups demonstrated a clinically meaningful improvement in MRC dyspnea scores following surgery (within-group p < 0.001). Postoperative dyspnea scores were significantly lower in the slide tracheoplasty group compared with the tracheal resection with end-to-end anastomosis group (median 0 vs 0–1; p < 0.05, r = 0.42).

A significant postoperative improvement in VHI-10 scores was observed in both groups (p < 0.001). Patients undergoing slide tracheoplasty achieved significantly lower postoperative VHI-10 scores compared with those treated with tracheal resection with end-to-end anastomosis (median 0 vs 10; p < 0.05, r = 0.38).

Preoperative GUSS scores were normal in all patients with no significant difference between groups. At one week postoperatively, GUSS scores were significantly lower in the tracheal resection with end-to-end anastomosis group compared with the slide tracheoplasty group (18 vs 15; p < 0.05). By one month, swallowing function recovered in both groups with no statistically significant difference.

**Fig. 4:**
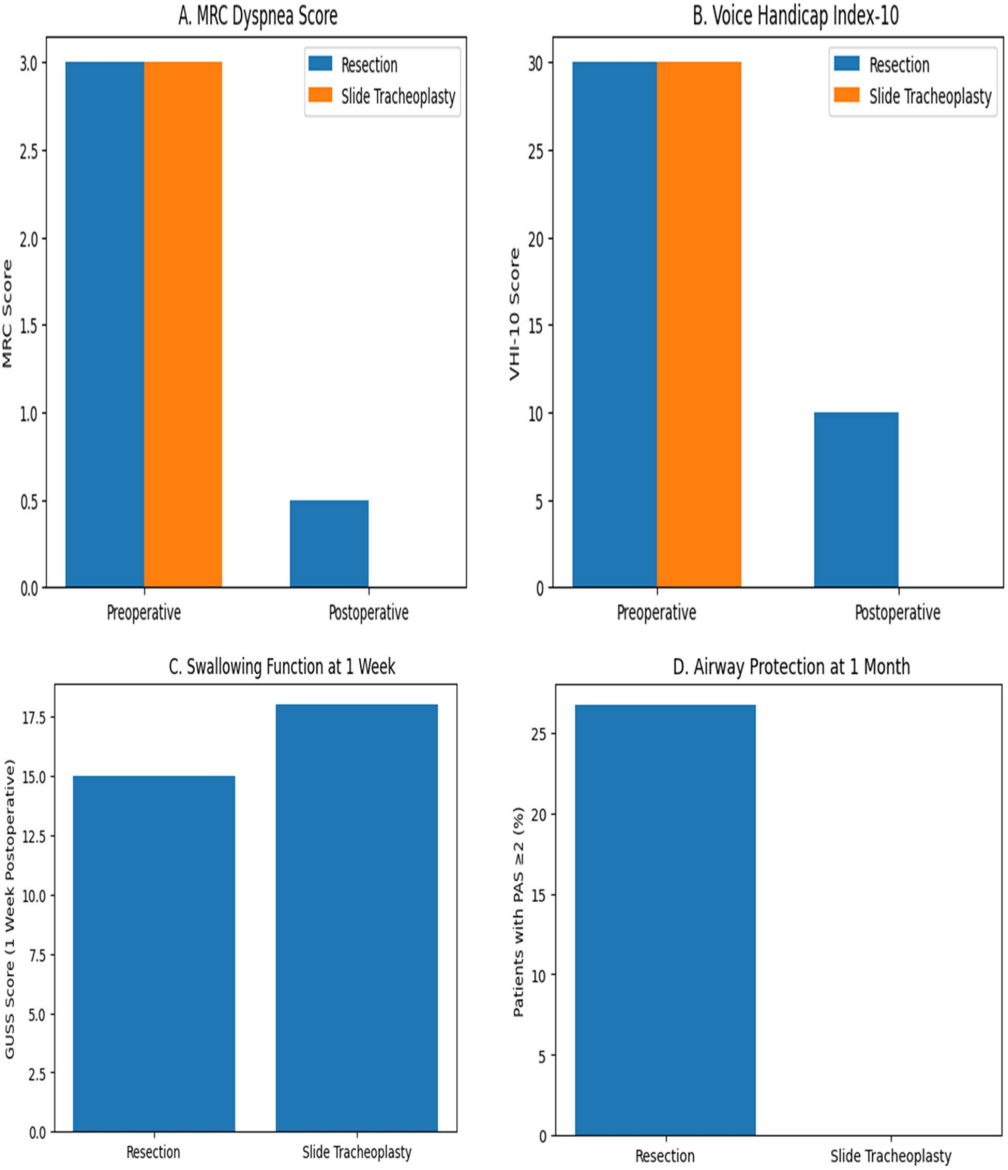
Functional Outcomes Following Airway Reconstruction**.** (**A**) Medical Research Council (MRC) dyspnea scores preoperatively and postoperatively in both groups. (**B**) Voice Handicap Index-10 (VHI-10) scores preoperatively and postoperatively. (**C**) Gugging Swallowing Screen (GUSS) scores at 1-week postoperative assessment. (**D**) Proportion of patients with Penetration–Aspiration Scale (PAS) ≥ 2 at 1-month postoperative evaluation.

All patients demonstrated normal airway protection preoperatively (PAS 1). At one month postoperatively, minor penetration (PAS 2–3), without documented aspiration events, was observed exclusively in the tracheal resection with end-to-end anastomosis group, whereas all patients in the slide tracheoplasty group maintained normal airway protection (p < 0.05) (Table 3), (Fig. 4).

**Table 3:** Functional Outcomes

Outcome measure	Resection (n = 15)	Slide tracheoplasty (n = 15)	p value	Effect size (95% CI)
MRC Dyspnea Scale				
Preoperative	3 (3–4)	3 (3–4)	NS	
Postoperative	0 (0–1)	0 (0–0)	< 0.05	r = 0.42
Within-group change	Significant	Significant	< 0.001‡	
VHI-10				
Preoperative	30 (20–40)	30 (30–40)	NS	
Postoperative	10 (0–10)	0 (0–10)	< 0.05	r = 0.38
Within-group change	Significant	Significant	< 0.001‡	
GUSS score				
Preoperative	20 (20–20)	20 (20–20)	NS	
1-week postoperative	15 (11–17)	18 (17–20)	< 0.05	r = 0.35
1-month postoperative	20 (18–20)	20 (20–20)	NS	
PAS score (FEES)				
Preoperative (PAS 1), n (%)	15 (100%)	15 (100%)	NS	
1-month postoperative (PAS ≥ 2), n (%)	4 (26.7%)	0 (0%)	< 0.05	

MRC = Medical Research Council; VHI-10 = Voice Handicap Index-10;

GUSS = Gugging Swallowing Screen; PAS = Penetration–Aspiration Scale;

NS = not significant.

## Discussion

**Fig. 5:**
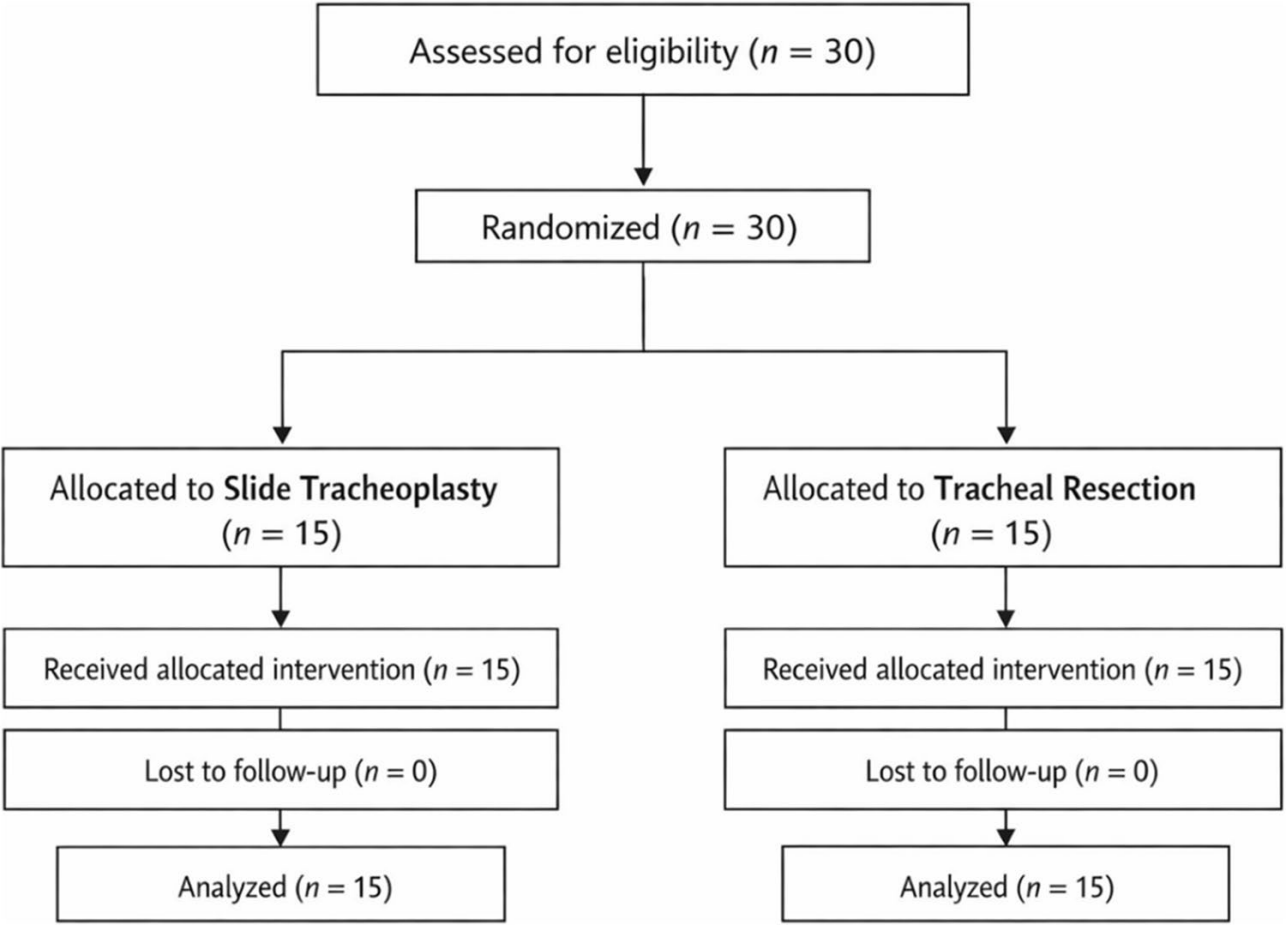
Consort flow chart showing study design

This pilot randomized controlled trial offers preliminary randomized evidence on two established surgical procedures for acquired laryngotracheal stenosis: slide tracheoplasty and tracheal resection with end-to-end anastomosis. The main goal of this feasibility study was to assess perioperative safety and surgical success while generating effect size estimates, rather than to establish definitive comparative superiority. Both techniques were associated with high rates of airway restoration with acceptable safety profiles and no mortality. Surgery-specific success exceeded 85% in both arms (93.3% in slide tracheoplasty and 86.7% in tracheal resection, supporting their continued use in appropriately selected patients. While slide tracheoplasty was associated with earlier decannulation and favorable trends in early functional recovery, these observations should be interpreted cautiously given the limited sample size and exploratory nature of the analysis [3–6, 11]. Fig. 5

The balanced baseline characteristics between the two groups strengthen the internal validity of the study and reduce the likelihood that observed differences are attributable to confounding factors or selection bias. Randomized allocation ensured comparable distributions of age, stenosis grade, etiology, stenosis length, and prior endoscopic interventions, allowing meaningful interpretation of postoperative differences as technique-related rather than patient- characteristics.

The definition of surgery-specific success in this study allowed up to two postoperative endoscopic dilatations without classification as failure. This approach highlights clinically meaningful airway stability rather than strict procedural definitions. This threshold is consistent with our institutional protocol, whereby failure to achieve airway stabilization after two dilatations indicates unsuccessful transluminal management. However, alternative definitions reported in the literature may yield lower success rates and could influence between-group comparisons [5–7]. Accordingly, the exploratory nature of this trial necessitates cautious interpretation of success outcomes.

The relatively young cohort reflects institutional referral patterns to a specialized airway reconstruction center, where both pediatric and young adult patients with acquired stenosis are managed. Although both age groups were treated according to the same surgical and perioperative protocols, anatomical and physiological differences may influence healing and recovery. This heterogeneity may limit generalizability to older adult populations, which represent the typical epidemiology of acquired laryngotracheal stenosis. Future studies should consider stratified or age-specific analyses to better define differential outcomes across age groups.

When interpreted in the context of existing literature, our findings are consistent with previously reported success rates for both procedures. Conventional tracheal resection with end-to-end anastomosis has demonstrated success rates of approximately 85–95%, while contemporary slide tracheoplasty series have reported similar outcomes [5–7]. The present study extends this evidence by providing a direct, randomized comparison within the same clinical setting, thereby strengthening the inference that slide tracheoplasty is not merely feasible but genuinely competitive with the historical tracheal resection with end-to-end anastomosis for acquired tracheal stenosis.

From a procedural standpoint, the two operations demonstrated distinct operative profiles. Slide tracheoplasty required longer operative time, reflecting its technical complexity, meticulous airway dissection and geometric reconstruction. However, this increase in operative duration did not translate into excess morbidity or prolonged hospitalization. Instead, the patients experienced earlier decannulation and a moderate reduction in hospital stay, both supported by moderate-to-large effect sizes. These findings suggest that operative time alone is a poor surrogate for postoperative recovery and that early functional gains may offset the added intraoperative complexity [12].

Single-stage reconstruction was achieved more frequently in the slide tracheoplasty group, although this difference did not reach statistical significance. Clinically, the ability to achieve single-stage airway reconstruction is highly desirable, as it reduces tracheostomy dependence and facilitates earlier rehabilitation [12]. The observed trend suggests that slide tracheoplasty may offer greater flexibility in achieving single-stage repair, without compromising safety, and an advantage that may become apparent in larger studies [5, 6].

Earlier decannulation in the slide tracheoplasty group represents one of the most clinically meaningful findings of this study. Reduced time to decannulation not only improves patient comfort and quality of life but may also decrease the risk of tracheostomy-related complications[12]. The requirement for postoperative balloon dilatation also showed a clinically relevant reduction in the slide tracheoplasty group. Although this difference did not reach statistical significance, the lower relative risk may represent a clinically meaningful advantage that could be confirmed in a larger adequately powered trial. These benefits are biologically plausible, as the sliding technique enlarges luminal diameter, preserves circumferential vascularity, and minimizes anastomotic tension, thereby promoting durable airway patency [13–15]. Overall complication and restenosis rates were comparable between the two techniques, and no unexpected adverse events or procedure-related harms occurred, further supporting the safety profile of slide tracheoplasty despite its technical complexity.

Beyond structural patency, functional recovery revealed clearer separation between techniques. Both procedures markedly improved dyspnea, yet patients undergoing slide tracheoplasty achieved lower postoperative MRC scores, indicating superior symptomatic relief. Voice outcomes followed a similar pattern, with better VHI-10 scores after slide repair. These differences likely reflect preservation of tracheal length, reduced laryngotracheal traction, and maintenance of a larger cross-sectional airway area compared with circumferential resection. Although these effects may not influence survival or overall patency, they represent clinically meaningful improvements in patient-reported outcomes [1, 2, 16].

Swallowing outcomes provided additional nuance. Early postoperative dysphagia occurred predominantly after tracheal resection with end-to-end anastomosis, whereas slide tracheoplasty preserved swallowing function more consistently. The transient impairment observed after tracheal resection with end-to-end anastomosis may relate to postoperative edema, neural traction, or altered laryngeal mechanics. Importantly, swallowing function recovered in both groups by one month, indicating no persistent dysphagia. Minor penetration events (PAS 2–3) were observed in a small subset of patients in the tracheal resection with end-to-end anastomosis group, while all patients in the slide tracheoplasty group maintained normal airway protection. These early functional differences, although transient, may influence postoperative rehabilitation, nutritional recovery, and patient-reported quality of life. [2, 9, 16, 17].

Taken together, the results suggest not superiority in absolute success but differing recovery trajectories. Tracheal resection with end-to-end anastomosis delivers dependable anatomical correction with shorter operative time, whereas slide tracheoplasty appears to promote earlier decannulation, fewer secondary interventions, and improved early functional outcomes. This distinction is clinically meaningful and suggests that procedure selection should consider not only stenosis characteristics but also rehabilitation priorities and patient-centered goals.

Given the small sample size and the use of exact statistical methods, several comparisons yielded p-values approaching unity and are better interpreted as indicating a lack of detectable difference rather than equivalence. Accordingly, effect size estimates provide more informative insights into potential group differences than p-values alone in this context. The results emphasize the importance of focusing on magnitude and direction of effects in pilot randomized trials, where statistical significance is not the primary objective.

To the best of our knowledge, this could be the first reported study in the literature to compare between slide tracheoplasty and tracheal resection with end-to-end anastomosis in in patients with acquired laryngotracheal stenosis being operated in the same center. However, some limitations could be identified. First, the pilot sample size limits statistical power for infrequent outcomes such as restenosis or reintervention, raising the possibility of type II error. Second, the single-center design may reflect institutional expertise and may not fully generalize to lower-volume settings; however, it also ensures procedural consistency and standardized perioperative care. Third, the inclusion of both pediatric and adult patients introduces heterogeneity that may influence outcomes. Fourth, follow-up was limited to six months; longer observation is necessary to assess durability of airway patency and functional benefits. Finally, the study was not powered for definitive comparative conclusions, and findings should be interpreted as hypothesis-generating. Nonetheless, the randomized design, complete follow-up, and standardized functional assessments strengthen the internal validity of these findings.

## Conclusion

This study provides preliminary evidence that both slide tracheoplasty and tracheal resection with end-to-end anastomosis are safe and effective surgical options for acquired laryngotracheal stenosis. Slide tracheoplasty, despite longer operative time, was associated with earlier decannulation and demonstrated a trend toward improved early functional recovery without an increase in complications. These findings suggest that slide tracheoplasty may represent a viable reconstructive option in selected patients. Larger multicenter randomized trials are required to confirm these findings, refine patient selection criteria, and establish definitive comparative effectiveness.

## Data Availability

Available from the corresponding author upon reasonable request.
